# Diagnostic Value of JC Polyomavirus Viruria, Viremia, Serostatus and microRNA Expression in Multiple Sclerosis Patients Undergoing Immunosuppressive Treatment

**DOI:** 10.3390/jcm11020347

**Published:** 2022-01-11

**Authors:** Carla Prezioso, Marco Ciotti, Gabriele Brazzini, Francesca Piacentini, Sara Passerini, Alfonso Grimaldi, Doriana Landi, Carolina Gabri Nicoletti, Maria Antonella Zingaropoli, Marco Iannetta, Marta Altieri, Antonella Conte, Dolores Limongi, Girolama Alessandra Marfia, Maria Rosa Ciardi, Claudio Maria Mastroianni, Anna Teresa Palamara, Ugo Moens, Valeria Pietropaolo

**Affiliations:** 1 IRCSS San Raffaele Roma, Microbiology of Chronic Neuro-Degenerative Pathologies, 00163 Rome, Italy; 2 Department of Public Health and Infectious Diseases, “Sapienza” University of Rome, 00185 Rome, Italy; gabriele.brazzini@uniroma1.it (G.B.); francescapiacentini.1854105@studenti.uniroma1.it (F.P.); passerini.1915659@studenti.uniroma1.it (S.P.); mariaantonella.zingaropoli@uniroma1.it (M.A.Z.); maria.ciardi@uniroma1.it (M.R.C.); claudio.mastroianni@uniroma1.it (C.M.M.); 3 Laboratory of Virology, Polyclinic Tor Vergata Foundation, 00133 Rome, Italy; marco.ciotti@ptvonline.it; 4 Multiple Sclerosis Clinical and Research Unit, Fondazione Policlinico di Tor Vergata, 00133 Rome, Italy; alfonso.grimaldi@uniroma2.it (A.G.); doriana.landi@gmail.com (D.L.); carolgabri@gmail.com (C.G.N.); marfia@uniroma2.it (G.A.M.); 5 Department of Systems Medicine, Tor Vergata University, 00133 Rome, Italy; marco.iannetta@uniroma2.it; 6 Department of Human Neurosciences, Sapienza University of Rome, 00185 Rome, Italy; marta.altieri@uniroma1.it (M.A.); antonella.conte@uniroma1.it (A.C.); 7 IRCCS Neuromed, 86077 Pozzilli, IS, Italy; 8 IRCCS San Raffaele Roma, Telematic University, 00163 Rome, Italy; dolores.limongi@uniroma5.it; 9 Unit of Neurology, IRCCS Istituto Neurologico Mediterraneo NEUROMED, 86077 Pozzilli, IS, Italy; 10 Department of Infectious Diseases, Istituto Superiore di Sanità, 00161 Rome, Italy; direzione.dmi@iss.it; 11 Department of Public Health and Infectious Diseases, Laboratory Affiliated to Institute Pasteur Italia-Cenci Bolognetti Foundation, Sapienza University of Rome, 00185 Rome, Italy; 12 Department of Medical Biology, Faculty of Health Sciences, University of Tromsø—The Arctic University of Norway, 9037 Tromsø, Norway; ugo.moens@uit.no

**Keywords:** progressive multifocal leukoencephalopathy, natalizumab, ocrelizumab, dimethyl-fumarate, fingolimod, JCPyV infection, non-coding control region

## Abstract

Markers of JC polyomavirus (JCPyV) activity can be used to evaluate the risk of progressive multifocal leukoencephalopathy (PML) in treated multiple sclerosis (MS) patients. The presence of JCPyV DNA and microRNA (miR-J1-5p), the anti-JCV index and the sequence of the non-coding control region (NCCR) in urine and plasma were determined in 42 MS subjects before treatment (T0), 6 months (T6) and 12 months (T12) after natalizumab, ocrelizumab, fingolimod or dimethyl-fumarate administration and in 25 healthy controls (HC). The number of MS patients with viruria increased from 43% at T0 to 100% at T12, whereas it remained similar for the HC group (35–40%). Viremia first occurred 6 months after treatment in MS patients and increased after 12 months, whereas it was absent in HC. The viral load in urine and plasma from the MS cohort increased over time, mostly pronounced in natalizumab-treated patients, whereas it persisted in HC. The archetypal NCCR was detected in all positive urine, whereas mutations were observed in plasma-derived NCCRs resulting in a more neurotropic variant. The prevalence and miR-J1-5p copy number in MS urine and plasma dropped after treatment, whereas they remained similar in HC specimens. Viruria and miR-J1-5p expression did not correlate with anti-JCV index. In conclusion, analyzing JCPyV DNA and miR-J1-5p levels may allow monitoring JCPyV activity and predicting MS patients at risk of developing PML.

## 1. Introduction

The arsenal of medications to treat multiple sclerosis (MS) has been enriched by a variety of drugs with differing mechanisms of action. Specifically, to date, 13 disease-modifying therapies (DMTs) for treatment of relapsing-remitting MS (RRMS) have been approved by the Food and Drug Administration (FDA), changing the scenery for RRMS patients’ care [1]. Although these drugs allow long periods of disease activity-free remission [2], they are correlated with a higher risk of side effects. Several DMTs have been associated with progressive multifocal leukoencephalopathy (PML), a demyelinating disease of the central nervous system (CNS) caused by a lytic infection sustained by JC polyomavirus (JCPyV), a circular double-stranded DNA virus with a restricted cellular tropism [3]. PML evolves in the context of an underlying immunological anomaly and in cell-mediated immunity deficiency, as demonstrated by more than 60% of PML cases occurring concomitantly with hematological malignancies [4] or in AIDS patients [5]. Natalizumab (Tysabri^®^) is one of the most effective therapies for the treatment of active RRMS [6] and, regardless its efficacy, long-term use (mostly more than 18 months) is associated with increased risk of PML [7]. The risk to develop PML with other DMTs is lower than with natalizumab, but it is not clear whether this is because of different mechanisms of action of these drugs or unsuitable screening methodology [8]. A global risk-management protocol for natalizumab includes assessment of anti-JCPyV antibody status prior to and during natalizumab treatment [9].

Currently, the risk-mitigation strategy developed for natalizumab is probably applicable only in relation to this drug. In fact, for example, under ocrelizumab treatment, in the clinical setting, an anti-JCPyV index as a *marker* of viral replication, which usually takes place in B lymphocytes reservoir, is commonly used. Since ocrelizumab exerts its effect by depleting B lymphocytes from peripheral circulation [10], monitoring the anti-JCV index may not be suitable for predicting individual risk of developing PML.

In the last two decades, many other candidate *markers* such as L-selectin (CD62L) [11], C-reactive protein or agnoprotein detection [12], T-cell response [13], JCPyV DNA detection in urine (viruria) and blood (viremia) [14,15,16,17] and modifications in the non-coding control region (NCCR) of JCPyV [15] have been proposed to predict the PML risk. Moreover, since JCPyV reactivation and PML onset involve the activation of the transcription of the viral genome [18], the quantification of viral RNA is another possible assay for determining viral activity.

A special class of transcripts are small non-coding transcripts known as microRNAs (miRNAs). These short transcripts regulate gene expression through translational repression and/or mRNA degradation, and consequently, aberrant miRNA expression is implicated in many diseases [19,20,21]. Cellular miRNAs can also be released in extracellular vesicles, and the levels of these extracellular miRNAs in biological fluids have become very valuable *markers* of malignant diseases and non-malignant pathologies such as Alzheimer’s disease and diabetes [22,23,24]. Moreover, miRNAs form therapeutic targets [25,26]. Several viruses encode their own sets of miRNAs, which can have self-regulatory or host modulating roles [21] and can also be released in extracellular vesicles by infected cells [27]. Some polyomaviruses, including JCPyV, encode their own unique microRNA that is produced as part of the late transcription in an infected host cell [28,29]. These microRNAs are thought to control viral replication through downregulation of the expression of large T-antigen, a viral protein absolutely required to replicate the viral genome. Reduced T-antigen expression results in downregulation of the host immune response and facilitation of persistent viral infection [30]. JCPyV encodes a primary miRNA (pri-miR-J1) in the LT-coding region of the viral genome but in an opposite sense [29]. The pri-miR-J1 is processed into a precursor miRNA (pre-miR-J1) that produces two mature miRNAs: miR-J1-5p and -3p [29]. The discovery of JCPyV miRNAs circulating in biofluids encouraged their use as diagnostic markers [31]. Indeed, JCPyV-encoded miRNAs have been identified in PML tissues [29], in multiple sclerosis patients undergoing natalizumab treatment [32], in HIV-infected patients [33], in cancer patients [34] and in healthy people [35]. Thus, the expression of miR-J1 could be related to the progression of PML, but since they were also detected in the blood and urine of patients without PML and of healthy individuals [29,33,36], the expression of miR-J1 in these body fluids could be unrelated to the progression of PML. Although the diagnostic potential of JCPyV miRNAs has been proposed for JCPyV infection in the gastrointestinal tract and other anatomical districts [34,37], the translational significance and clinical value of these miRNAs remain to be determined.

In the light of these consideration, we evaluated the diagnostic value of JCPyV viruria, viremia, serostatus and microRNA expression in multiple sclerosis patients undergoing immunosuppressive treatment to estimate the feasibility of using these different *biomarkers* for JCPyV infection and for PML.

## 2. Results

### 2.1. Longitudinal Investigation of JCPyV Viruria and Viremia in MS Patients

One urine sample and one plasma sample from 42 subjects diagnosed with MS obtained before starting treatments (=T0) were tested for the presence of JCPyV DNA. Overall, JCPyV DNA was detected in 18/42 (43%) urine samples. Specifically, viral DNA with a viral load mean value of 5.5 × 10^2^ copies/mL was detected in 6/19 urine specimens from patients to be given ocrelizumab and in 10/19 urine samples with a JC viral load mean value of 5 × 10^3^ copies/mL obtained from patients to be treated with natalizumab. One patient of the two subjects who were candidates for treatment with fingolimod and one patient of two subjects to be treated with dimethyl-fumarate (DMF) had a JCPyV-DNA-positive urine sample with a JC viral load of 3.5 × 10^3^ copies/mL and of 3.0 × 10^3^ copies/mL, respectively. None of the 42 patients had detectable JCPyV DNA in their plasma samples before treatments were initiated (T0). After six months of treatment administration (T6), JCPyV DNA was detected in 30/42 (71%) urine and in 3/42 (7.1%) plasma samples. Viral DNA was detected in 10 out of 19 urine specimens from ocrelizumab-receiving patients, in 18 out of 19 urine samples from patients treated with natalizumab and in urine from the same fingolimod and DMF patient’s samples that were positive for JCPyV DNA at T0. Results of qPCR at T0 and T6 revealed a 2-fold increase in viral load mean value (5.5 × 10^2^ versus 1 × 10^3^ copies/mL) in patients treated with ocrelizumab, > 100-fold (5 × 10^3^ versus 7 × 10^5^ copies/mL) in patients in who natalizumab was administrated and > 10-fold increase (3.5 × 10^3^ versus 5.5 × 10^4^ copies/mL and 3.0 × 10^3^ versus 5.0 × 10^4^ copies/mL) in patients treated with fingolimod and DMF. In 3 out of 19 natalizumab patients in whom JCPyV DNA was detected in urine samples, viral DNA was also present in plasma, with a viral load mean value of 1.5 × 10^3^ copies/mL. None of the other patients had detectable JCPyV viremia (Table 1).

Finally, 12 months after the beginning of therapy (T12), all patients presented JCPyV DNA in urine (42/42, 100%), and among these, six patients were also viremic. Analysis of qPCR performed on urine samples showed a further increase in mean of viral load that was of 6 × 10^4^ copies/mL in patients treated with ocrelizumab, of 8.5 × 10^6^ copies/mL in patients treated with natalizumab and of 1.5 × 10^5^ copies/mL and 1.0 × 10^5^ copies/mL in patients treated with fingolimod and DMF, respectively (Table 1).

Regarding the viral plasmatic presence, JCPyV DNA detection was confirmed in the three natalizumab patients in whom viremia was just observed at T6, with a mean of viral load of 2.0 × 10^5^ copies/mL, and in plasma samples of three other patients: one ocrelizumab patient (mean value of 1 × 10^3^ copies/mL), one patient receiving fingolimod (mean value of 5.5 × 10^3^ copies/mL) and one patient under DMF administration (mean value of 7.5 × 10^3^ copies/mL) (Table 1).

The detection of JCPyV DNA was also performed throughout the study (T0, T6 and T12) in urine and plasma samples from the 25 HCs population. Results showed viral shedding only in urine from 10/25 (40%) HCs at T0, with a mean viral load of 2.5 × 10^3^ copies/mL, 8/25 (32%) HCs at T6 with a mean viral load of 5 × 10^3^ copies/mL and in 9/25 healthy subjects (36%) at T12 with a mean viral load of 3 × 10^3^ copies/mL (Table 2). We found no subjects with detectable JCPyV DNA in plasma, similar to previous observations [36,38].

### 2.2. Non-Coding Control Region (NCCR) Analysis

Because rearrangements in the NCCR are associated with the pathogenesis of JCPyV [15], we sequenced the NCCR in all virus-positive urine and plasma samples. As expected, the JCPyV NCCR analysis performed from T0 to T12 showed a structural organization identical to archetypal JCPyV NCCR (i.e., A-B-C-D-E-F box arrangement) in all analyzed positive urine samples. The NCCR analysis performed at T6 on the three positive plasma samples detected in natalizumab patients displayed mutations throughout the NCCR sequence. Specifically, the first plasmatic NCCR analyzed was characterized by a duplication of NF-1 cellular transcription factor binding site (box F) and by a truncated box D composed only of 10 out of 65 bases (nucleotides from 117 to 126). The second JCPyV NCCR sequenced revealed the duplication of box C and the deletion of nucleotides from 117 to 180, corresponding to box D. Finally, the third plasma-derived JCPyV NCCR studied showed a partially deleted C-box (first 38 nucleotides were maintained out of 55). At T12, the first and the second NCCR isolated from plasma of natalizumab patients showed the same structure observed at T6, whereas the third plasmatic NCCR, in addition to the mutations described after 6 months of natalizumab administration, presented a complete deletion of the D-box and a partial deletion of the E- and the F-box (the last 9 nucleotides out of 18 in E-box and the first 5 nucleotides out of 69 in F-box were maintained). After one year of ocrelizumab administration (T12), the NCCR isolated from one plasma sample presented the transversion 37T → G in the Spi-B binding site (box B). The NCCR structure isolated of one plasma sample from a patient under fingolimod administration and of one plasma sample from a patient treated with DMF showed the transversion 37T → G in the Spi-B binding site (box B), together with the transition 217G →A in the box F and the duplication of the nucleotides from 54 to 57 in the B-box, from 60 to 85 in the C-box and from 189 to 196 in the E-box, respectively.

The NCCR sequence was also determined in the virus-positive samples from the individuals in the HC group. As expected, the NCCR structure showed an organization identical to archetype JCPyV in all analyzed positive urine samples.

### 2.3. The Anti-JCV Index

During the study’s observation period, the anti-JCV index value pre- and post-initiation of treatment administration was also measured (T0, T6 and T12). At T0, 0/42 patients had high risk of PML (anti-JCV index > 1.5), 2 patients (2/19 subject to be treated with natalizumab) presented a medium risk of PML (0.9 < anti-JCV index > 1.5) and 40/42 patients (19/19 who would be treated with ocrelizumab, 17/19 with natalizumab and 4/4 with fingolimod and DMF) had a low risk of PML (anti-JCV index < 0.9) (Table 1). At T6 the number of patients presenting an anti-JCV index > 1.5 increased to 19/42 (5/19 ocrelizumab, 12/19 natalizumab and 1/2 fingolimod and 1/2 DMF). At T6, 13 patients had an anti JCV index 0.9 < JCV index > 1.5. Of these, were 5/19 receiving ocrelizumab, 6/19 were treated with natalizumab, 1 under fingolimod and 1 under DMF administration. Finally, 10/42 patients (9 under ocrelizumab and 1 treated with natalizumab) possessed an anti-JCV index < 0.9 (Table 1). At T12, patients who had high risk of PML (anti-JCV index > 1.5) included 10/19 ocrelizumab, 15/19 natalizumab, 1/2 fingolimod and 1/2 DMF. A total of 11 patients presented a medium risk of PML (0.9 < JCV index > 1.5), of whom were 7 ocrelizumab-, 2 natalizumab-, 1 fingolimod- and 1 DMF-treated subjects. Finally, 4/42 patients with a low risk of PML (anti JCV index < 0.9) were identified (2/19 ocrelizumab and 2/19 natalizumab) (Table 1). The anti-JCV index was not available for HCs.

### 2.4. Combined Monitoring of the Serostatus and Its Relationship to Viral DNA

**Figure 1:**
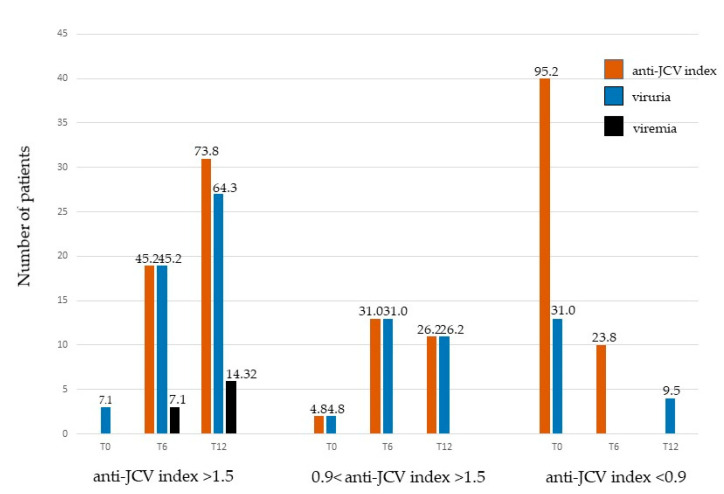
Relationship between serostatus and JCPyV DNA in 42 individuals with MS. T0: before starting treatments, no drug administration; T6: 6 months after the beginning of therapy; T12: 12 months after the beginning of therapy. The number on top of each bar indicates the percentage of MS persons with anti-JCV index, viruria and viremia, respectively. The absence of a bar indicates that none of the individuals with MS had the indicated anti-JCV index, viruria and viremia, respectively.

Although none of the patients had an anti-JCV index > 1.5 at T0, three patients showed viruria, and these were patients enrolled to be administered ocrelizumab (3/19). At T6, 19 patients had an anti-JCV index > 1.5, and JC viruria was detected in each of these patients (ocrelizumab: 5/10, natalizumab: 12/18, fingolimod: 1/2, and DMF: 1/2). It is worth noting that after 6 months of drug administration, three natalizumab patients with an anti-JCV index > 1.5 shed JC viral DNA concomitantly in urine and in plasma. At T12, among the 31/42 patients showing an anti-JCV index > 1.5, viral DNA was detected concomitantly in 10/19 ocrelizumab-, 15/19 natalizumab-, 1/2 fingolimod-, and 1/2 DMF-treated patients. At T12, one patient treated with ocrelizumab, three patients treated with natalizumab, one patient under fingolimod, and one DMF-treated patient with an anti-JCV index >1.5 presented viremia in addition to viruria (Figure 1).

Only two patients had a medium risk of PML (0.9 < JCV index > 1.5) at T0. Both belonged to patients to be treated with natalizumab, and they excreted JCPyV DNA in urine.

At T6, 13/42 patients presented an anti-JCV index between 0.9 and 1.5 and concomitantly had JCPyV viruria. Of these, five were patients treated with ocrelizumab, six with natalizumab, one with fingolimod and one with DMF. Analysis conducted after one year of drug administration (T12) revealed that there were 11 patients with an anti-JCV index between 0.9 and 1.5. Among these, viral DNA was observed in seven treated with ocrelizumab, two treated with natalizumab, one with fingolimod and one with DMF (Figure 1).

To conclude, 40/42 patients had an anti-JCV index ≤ 0.9 at T0, and among these, 13 patients also displayed viruria (3 patients who would be treated with ocrelizumab, 8 with natalizumab, 1 patient with fingolimod and 1 with DMF). At T6, 10/42 patients possessed an anti-JCV index ≤ 0.9. Of these, nine were under ocrelizumab administration and one under natalizumab treatment. None of these excreted JCPyV DNA in the urine. None of the patients showed an anti-JCV index ≤ 0.9 at T12, although JCPyV viruria was detected in two patients under ocrelizumab treatment and in two patients to whom natalizumab was administrated (Figure 1).

### 2.5. JCPyV miRNAs Investigation

Increased levels of JCPyV miR-J1-3p and miR-J1-5p have been detected in blood samples from MS patients under natalizumab treatment [32]. The presence of miR-J1-3p and miR-J1-5p miRNAs was investigated in each sample obtained from 42 MS patients (42 urine and 42 plasma specimens collected at T0, at T6 and at T12) and from 25 HCs (25 urine and 25 plasma samples for a total of 50 specimens for each time point). Overall, at T0, miR-J1-5p was detected in 26/42 (62%) urine samples from 15/19 patients enrolled to be given ocrelizumab, from 9/19 patients who would be treated with natalizumab and from 2 patients who were candidates for treatment with fingolimod. qPCR results showed a mean expression level of 1000 copies/ng of RNA for miR-J1-5p. Analysis of plasma samples demonstrated the expression of miR-J1-5p in 27/42 (64%) samples. Twenty-six of these were from the same patients whose urine samples were also miR-J1-5p-positive, whereas the remaining sample belonged to a patient candidate for treatment with DMF. The mean expression level of miR-J1-5p was 900 copies/ng of RNA (Table 1). After six months of treatments administration (T6), miR-J1-5p was detected in 23/42 (55%) urine and in 23/42 (55%) plasma samples of the same patients. Specifically, miR-J1-5p was revealed in urine and plasma samples of patients treated with ocrelizumab (15/19), natalizumab (7/19) and fingolimod (1/2). Results of qPCR revealed a decrease in miR-J1-5p mean value to 800 copies/ng in urine samples and to 700 copies/ng in plasma samples (Table 1). Finally, 12 months after the beginning of therapy (T12), miR-J1-5p was detected both in urine and in plasma from 12/19 ocrelizumab patients, from 5/19 natalizumab patients and from 1/2 patient under fingolimod treatment, for a total of 18/42 patients (43%). Analysis of qPCR showed a further decrease in mean of miR-J1-5p expression that reached 500 copies/ng in urine and 540 copies/ng in plasma (Table 1).

Throughout the study (T0, T6 and T12), the detection of miR-J1-5p was also performed on urine and plasma samples from HCs. At T0, miR-J1-5p was detected in urine from 8/25 (32%) and in plasma from 16/25 (64%) healthy subjects. The mean value of the qPCR results was 800 copies/ng of RNA for miR-J1-5p in urine and 1000 copies/ng of RNA for miR-J1-5p in plasma samples. At T6, the number of urine samples in which miR-J1-5p was detected remained the same as observed at T0 (8/25), whereas the number of miR-J1-5p-positive plasma samples decreased from 16 to 15. The mean value of qPCR experienced a reduction (550 copies/ng of RNA for miR-J1-5p in urine and of 600 copies/ng of RNA for miR-J1-5p in plasma samples). After one year from the beginning of the study (T12), the number of samples (urine and plasma) in which miR-J1-5p was revealed was the same as the number observed at T0 (8/25 urine; 16/25 plasma). qPCR showed a miR-J1-5p expression with the same trend obtained at T0 (Table 2).

True positive miRNA-J1-3p signals were not detected in urine and plasma samples indisputably. In fact, only occasional amplification, with a very number of low copies, was obtained in urine samples of two MS patients and of three HCs. Since only one HC plasma sample gave a positive signal with acceptable copies/ng of RNA (200 copies/ng), the assessment of miRNA-J1-3p expression in MS patient groups and in HCs group was not performed.

### 2.6. Combined Assessment of JCPyV DNA Detection, Serostatus and miRNA Expression

**Figure 2:**
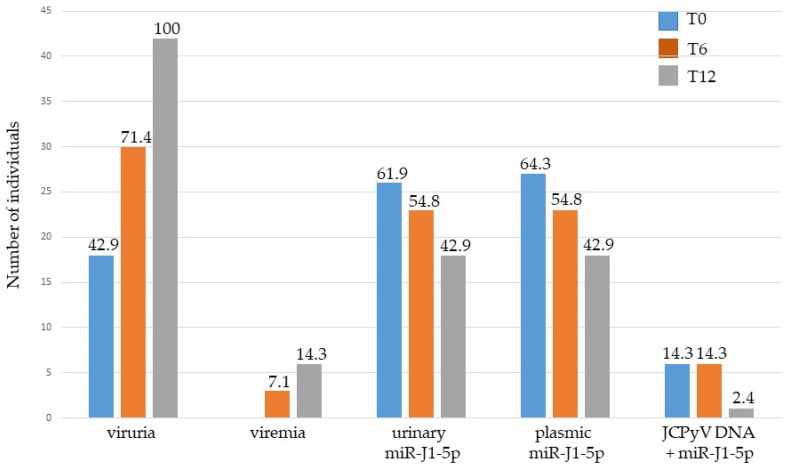
Combined assessment of JCPyV DNA detection, serostatus and miRNA expression in 42 subjects with MS. T0: before starting treatments, no drug administration; T6: 6 months after the beginning of therapy; T12: 12 months after the beginning of therapy. The number on top of each bar represents the percentage of persons with MS who had viruria, viremia, urinary or plasmic miR-J1-5p or concomitant JCPyV DNA and miR-J1-5p detection, respectively.

As described above, at T0, JCPyV DNA was found in 18/42 urine samples belonging to MS patients. Analyzing the miRNA expression, miR-J1-5p was detected in 26/42 urine and in 27/42 plasma samples. Concomitant presence of JCPyV DNA and miR-J1-5p expression showed that of the 18/42 patients with viruria only 6 patients expressed miR-J1-5p. After 6 months’ treatment (T6), JCPyV DNA was detected in 30/42 urine samples and in 3/42 plasma samples (Figure 2). Inverse to the increase in the number of patients who tested positive for viral DNA, the number of patients in which the expression of miR-J1-5p was revealed in urine decreased from 26 (T0) to 23 (T6), and in plasma from 27 (T0) to 23 (T6). Among these, JC viruria and viremia were simultaneously detected, with miR-J1-5p expression in five patients and in one patient, respectively (Figure 2). At T12, a reverse trend was established: JCPyV DNA was detected in all urine specimens and in 6/42 plasma samples (Figure 2), whereas the number of miR-J1-5p-positive samples showed a further decrease (from 23 to 18, both for urine and plasma). JCPyV DNA, in addition to miR-J1-5p expression, was confirmed in the same one viremic patient observed at T6 (Figure 2). 

The JC viral load mean value and the mean expression level of miR-J1-5p were also evaluated. As the number of patients who excreted viral DNA in urine and plasma increased during the study, the mean numbers of JCPyV DNA copies/mL in urine and in plasma samples also increased over time (T0, T6 and T12), although with a logarithm of difference between the two types of biological samples. Conversely, analysis of qPCR performed on positive urine and plasma miRNA samples showed a decrease from T0 to T12 in the mean of miR-J1-5p expression. The expression level was lower in JCPyV-DNA-positive patients compared with the JCPyV-DNA-negative patients (*p* > 0.05) (Table 1).

Based on current medications, a significant difference was found between the miR-J1-5p expression levels found in natalizumab-treated patients and those found in patients treated with ocrelizumab, fingolimod and DMF (*p* = 0.030). Although no statistical significance was found between miR-J1-5p expression levels in fingolimod- and DMF-treated patients, no conclusions can be drawn since these groups of patients were too small (*p* > 0.05).

Finally, the association between miR-J1-5p expression and anti-JCV index was also studied. Among all treated patients, miR-J1-5p expression did not correlate with anti-JCV index. The levels of miR-J1-5p expression differed between the high-risk group and low-risk group of developing PML since it was higher among groups with low risk and lower among patients with an anti-JCV index > 1.5 (Table 1).

Viral shedding and detection of JCPyV miRNA was compared in HCs. Specifically, subjects in whom miR-J1-5p was detected in plasma (T0: 16/25, T6: 15/25 and T12: 16/25) did not reveal JCPyV DNA in the same analyzed samples (T0: 0/25, T6: 0/25 and T12: 0/25), whereas, when JCPyV DNA was detected in urine (T0: 10/25, T6: 8/25 and T12: 9/25), the subjects in whom miR-J1-5p was observed decreased (T0: 8/25, T6: 8/25 and T12: 8/25) (Table 2). The anti-JCV index was not available for HCs, and therefore, we were not able to correlate miRNA detection rates with the anti-JCV index among HCs.

### 2.7. JCPyV miRNA Region Sequences

**Figure 3:**
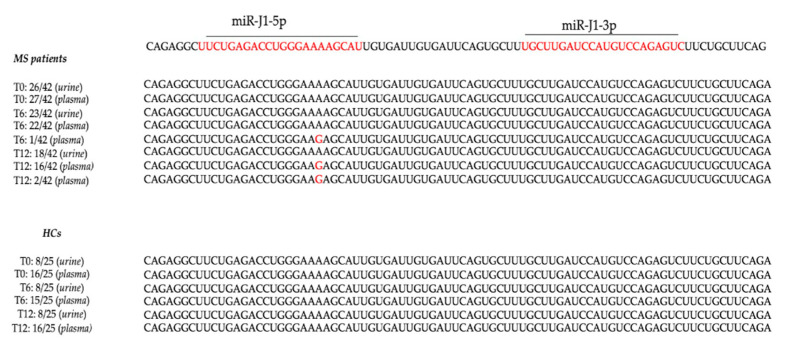
The miR-J1-5p mutations observed in JCPyV-DNA-positive MS specimens and in HCs. The mature miR-J1-3p and -5p are indicated (red color) by the bar over the sequence. The target region is indicated under the sequence. The JCV-miR-J1 sequence from miRBase V20 was used as a reference sequence [28]. MS patients: multiple sclerosis patients. HCs: healthy control subjects. T0: before starting treatments, no drugs administrated; T6: 6 months after the beginning of therapy; T12: 12 months after the beginning of therapy. The miR-J1-5p miRNA was investigated in each sample obtained from 42 MS patients. The positive samples, in which miR-J1-5p sequence was detected, are reported: at T0, miR-J1-5p was detected in 26/42 urine samples (62%) and in 27/42 (64%) samples. All analyzed samples presented a sequence identical to the miRBase V20 reference sequence [28]. At T6, miR-J1-5p was detected in 23/42 (55%) urine and in 23/42 (55%) plasma samples. A total of 23 urine and 22 plasma samples presented a sequence identical to the reference strain [28], whereas 1 plasma sample belonging to a MS patient treated with natalizumab displayed the transition A to G. At T12, miR-J1-5p was detected both in urine and in plasma from 18/42 patients (43%), and except for two plasma samples belonging to natalizumab-treated patients in which the transition A to G was observed, all sequences were identical to the reference strain [28]. Throughout the study (T0, T6 and T12), the detection of miR-J1-5p was also performed on urine and plasma samples of the HCs population. At T0, miR-J1-5p was detected in urine from 8/25 (32%) and in plasma from 16/25 (64%) HCs. At T6, the number of urine samples in which miR-J1-5p was detected remained the same as observed at T0 (8/25), whereas the number of miR-J1-5p-positive plasma samples decreased from 16 to 15. At T12, the number of urine and plasma in which miR-J1-5p was revealed was the same as the number observed at T0 (8/25 urine; 16/25 plasma).

To investigate JCPyV miRNA variability, the miRNA-encoding region found in all the JCPyV-DNA-positive urine and plasma samples was sequenced. In Figure 1 the observed mutations are reported. In particular, mutations were detected in plasmic miRNA (T6) from one natalizumab patient and in plasmic miRNA (T12) from two patients treated with the same drug. These sequences showed the presence of different changes compared with those obtained from MS patients under treatment with ocrelizumab, fingolimod and DMF and from HCs subjects (Figure 3).

## 3. Discussion

When considering medication for MS patients, risk of PML remains a major challenge for clinicians because, in addition to natalizumab, other MS treatments such as fingolimod, DMF or ocrelizumab have also been reported to be related to JCPyV reactivation and PML onset [39]. This has led to the need for reliable and sensitive prognostic markers to better manage on an individual basis the patients at risk of developing PML. In the last two decades, several studies have been carried out to identify sensitive and predictive markers detectable to prevent PML disease, including L-selectin (CD62L), C-reactive protein or agnoprotein detection, JCPyV serostatus, viremia, viruria and the onset of JCPyV NCCR variants. Moreover, since JCPyV reactivation and PML onset involve the activation of transcription of the viral genome [18], the quantification of viral RNA, including JCPyV miRNAs, is considered another possible assay for viral activity [29].

Considering this background, the purpose of this study was to evaluate different markers of JCPyV infection, enrolling a population composed of MS patients treated with different DMTs. In addition to JC viruria, viremia and serostatus, we investigated whether JCPyV-encoded miRNAs could be detected and whether the presence of these miRNAs could be related to serostatus or viremia and viruria. The same investigations were carried out on urine and plasma samples from HCs.

Regarding the analysis of JCPyV viruria, our results show that JC viral DNA was detected in urine of MS patients during the whole period of the study (T0, T6 and T12). Viral shedding was also detected in urine samples of HCs subjects throughout a one-year observation period. Specifically, at T0, i.e., before starting MS treatments, viruria was displayed at a similar detection rate between MS patients (43%) and HCs (40%), confirming that JC virus can be found in the urine of one third of healthy adults [40] and that JCPyV shedding may occur independently from the administration of different immunomodulatory medications, or in the presence of several immunocompromising factors related to their underlying disease. The number of HC individuals excreting JCPyV DNA in urine remained almost the same at T6 (32%) and T12 (36%), whereas, inverse to T0, the percentage of subjects affected by MS and under DMTs with viruria increased from 71% at T6 to 100% at T12. This finding is in line with the consistent observation for which DMTs might predispose to JCPyV reactivation and to the increment of viral replication [41]. Viremia was observed in MS patients after 6 and 12 months of DMTs administration but not in healthy subjects. These results emphasize that viremia followed the increase in viruria and that the initial site of viral reactivation is the kidney, where the decreased immunosurveillance caused by DMTs led to a secondary spread of virus and the subsequent release of JCPyV into the bloodstream [40]. Moreover, our data confirm that although JCPyV is found in urine of immunocompetent individuals, the frequency of JC viruria increases in the setting of immunosuppression, and usually, it is not detected in the blood of healthy persons or of untreated patients with multiple sclerosis [40].

Considering that JC viral load was monitored during the study, a progressive increment from T0 to T12 was observed, analyzing urine-positive samples from patients treated with all categories of treatments. An increase of two logarithms (from T6 to T12) was also observed analyzing the viral load of positive plasma samples belonging to three natalizumab patients. The increment in JC viral load in patients treated with natalizumab was significantly higher compared with those observed in patients under ocrelizumab, fingolimod or DMF (*p* < 0.05). Our results corroborate that natalizumab therapy strongly influences the viral ability to replicate, increasing the rate of JCPyV urinary and plasmatic shedding with respect to other drugs [40].

Since a higher JC viruria could correlate with viremia and the onset of NCCR variants, monitoring the trend of JC viral load could be a useful alert to also identify mutations and/or NCCR reorganization. The analysis of JCPyV NCCR presented the regulatory region with a stable and nonpathogenic archetypal pattern in all MS patients’ urine samples throughout the study, while the NCCR analysis performed on patients in whom viremia developed during natalizumab and ocrelizumab therapy displayed a neurotropic form of the JCPyV NCCR. Specifically, the duplication of NF-1 transcription factor binding site is well known to stimulate JCPyV’s early and late gene expression and to promote the onset of JCPyV variants with determinants of neurotropism and increased neurovirulence [42]. Moreover, the duplication of box C results in an extra copy of the cAMP response element binding site, a specific enhancer of JCPyV replication [43]. In this rearranged form it is significant to also observe the loss of the D-box that, as is already known, could represent one of the early but crucial steps in the complex series of NCCR rearrangements leading to PML. Finally, the transition such as 217G → A in the Spi-B binding site of box F was detected in a patient treated with ocrelizumab, and as previously reported, it could precede NCCR re-organization, and it is able to transform an archetype Spi-B binding site in a JCPyV PML-variant [44].

In our study, we confirmed that the NCCR box duplications occur often near to the origin of genome replication (upstream or 5′ of box A to C), whereas deletions were more frequent near to late genes (downstream or 3′ of box D to F) [42,45]. Duplications in the proximal part of the NCCR have been demonstrated to stimulate viral replication and early gene transcription, whereas deletions near the late region that removed control sequence repressed late transcription [42,45]. Although statistically not significant, mutations occurred with a higher percentage in natalizumab-treated patients (83.3%) than in ocrelizumab-treated patients at T12 (33.3%).

In this study, the reliability of anti-JCPyV antibodies as marker of JCPyV infection was also evaluated, and a contextual analysis of anti-JCPyV antibodies versus JCPyV DNA was carried out. The combined monitoring showed that although second-generation STRATIFY JCV explains an increased sensitivity, an anti-JCPyV antibodies assay alone is not sufficient for JCPyV diagnosis because some patients with low risk (≤0.9) displayed viruria. However, the viral load correlated well with the anti-JCV index because urine from patients with a higher anti-JCV index had a higher viral load compared with patients with lower anti-JCV index. Hence, our results indicate that the copy number of JCPyV DNA is a useful parameter for stratifying the PML risk since the detection of viruria identifies JCPyV-infected subjects when antibodies are still undetectable [46,47].

In this study, the presence of JCPyV-encoded miRNA in urine and plasma samples from MS patients treated with different DMTs and from HCs subjects was considered as another different measure of JCPyV infection. Specifically, JCPyV miRNA detection and its presence related to serostatus, viremia and viruria were evaluated.

Our longitudinal study showed that miR-J1-5p prevalence in urine and plasma from HCs remained, respectively, ~30% and ~60% during the observation periods T0, T6 and T12, whereas the copy number varied between 550 and 800 for urine specimens and 600 and 1000 for the plasma specimens. The lowest numbers were observed at T6. For MS patients, miR-J1-5p prevalence in urine and serum decreased over time. At T0, HC and MS patients had similar miR-J1-5p prevalence (64%), but prevalence decreased to 43% in plasma from MS patients. Urinal miRNA was observed in 62% of the MS patients at T0 but dropped to 43% at T12. The miR-J1-5p copy number reduced by almost 50% in both urine and serum at T12. Thus, treatment may stimulate viral replication by reducing miR-J1-5p, which downregulates expression of large T-antigen, a protein absolutely required for viral replication [31]. The concomitant analysis between shedders and non-shedders and miR-J1-5p evidenced that miR-J1-5p expression was inversely correlated with JCPyV detection. An inverse correlation was also confirmed by analyzing the mean value of JCPyV DNA copies/mL and the mean number of miR-J1-5p copies in all urine and plasma. This mechanistic model in which an increased level of viral miRNAs is associated with a lower viral load, and vice versa, could confirm an important role of JCPyV miRNA in controlling viral replication through downregulation of large T-antigen expression. Although to be studied further, the reduction in JCPyV miRNA expression in JCPyV-DNA-positive samples, related to an impairment in miR-J1-5p expression implicated in early viral reactivation, might be of clinical utility in immunocompromised patients whose JCPyV infectious status is being monitored.

The investigation of JCPyV 5p miRNA level in relation to serostatus pointed out that miRNA could also be detected in urine or plasma samples from JCPyV subjects with an anti-JCV index (<0.9), indicating that a negative JCPyV Ab result does not necessarily mean the absence of JCPyV infection [36] but could support a persistent/latent viral condition.

To conclude, our data emphasize that drugs affect JCPyV miRNA expression and confirm that natalizumab, more than other drugs, upon viral reactivation, increased JCPyV replication that could be accompanied by modifications within the JCPyV NCCR.

Although only two patients receiving fingolimod and two patients who received DMF were included in this study, in accordance with ocrelizumab- and natalizumab-treated patients, treatment with these two drugs changed the anti-JCV index from <0.9 to a higher index. Likewise, fingolimod and DMF administration over time resulted in viruria and viremia in some or all of the patients, with viral loads comparable or higher than the other two patient groups. A drop in copies of urinal and plasmic miR-J1-5p observed in the MS patients receiving ocrelizumab and natalizumab was also detected in fingolimod and DMF treated patients. Finally, mutations in the NCCR also occurred in patients receiving these two different treatments. Although the same tendency in response (anti-JCV index, viruria, viremia, urinary and plasmic miR-J1-5p shedding and mutations in the NCCR) was observed for the fingolimod and DMF patients, our study included only two of each group, and therefore, it is impossible to draw solid conclusions for these patient groups.

## 4. Materials and Methods

### 4.1. Study Participants and Samples Collection

This study included a total of 42 subjects diagnosed with MS, of whom 19 were treated with ocrelizumab, 19 patients were treated with natalizumab, 2 were treated with fingolimod, and 2 with dimethyl-fumarate (DMF).

MS patients were enrolled from January 2016 to May 2020. All patients underwent clinical and neurological examinations before sampling. The diagnosis of MS was based on the revised McDonald Criteria [48]. Neurological disability was evaluated by the Expanded Disability Status Scale (EDSS) score [49]. No cases of PML developed among the patients enrolled in this study. The study was approved by the Ethics Committee of the Independent Ethic Committee of Policlinico Tor Vergata (protocol number 8473/2019) and of Policlinico Umberto I (protocol number 130/13), and all subjects gave informed consent.

From the enrolled patients, 42 plasma and 42 urine samples were obtained before starting treatments (baseline T0: 0 administration) and after the beginning of therapy at the following time points: T6, 6 months, and T12, 12 months, for a total of 252 specimens. Patients were stratified according to their anti-JCV Ab index into groups with low risk of developing PML (anti-JCV index < 1.5), intermediate (0.9 < JCV index > 1.5) and high (anti-JCV index > 1.5) [50].

The study was also performed on 25 healthy control (HC) subjects enrolled to evaluate the utility of miRNAs in PML risk assessment. Urine and serum samples of each healthy individual were obtained at three different time points (T0, T6 and T12).

MS patients and HCs demographics and disease characteristics are reported in Table 3.

### 4.2. JCPyV Viruria and Viremia Investigation

JCPyV DNA was extracted using DNeasy Blood and Tissue Kit (Qiagen, Milan, Italy). Extraction products were analyzed by a quantitative PCR (Q-PCR) system able to detect a 54 bp amplicon in JCPyV T antigen region, using a 7300 real-time PCR system (Applied Biosystems, Waltham, MA, USA) [16,51,52]. Each sample was analyzed in triplicate, and JCPyV DNA loads (given as the mean of at least three positive reactions) were expressed as genome equivalents (gEq)/milliliter. Negative and positive controls were included in each Q-PCR session. Standard curve was obtained from serial dilutions (range: 10^5^–10^2^ gEq/mL) of a plasmid containing the entire JCPyV genome. The lower detection limit of the Q-PCR system was 10 DNA copies of the target gene per amplification reaction, corresponding to 10 genome equivalents per reaction (10 gEq/reaction). JCPyV-DNA-positive samples were further analyzed using nested-PCR for NCCR regions’ amplification [16]. PCR products were analyzed on 2% agarose gels. The amplified products were purified using MinElute PCR Purification Kit (Qiagen, Milan, Italy) and sequenced in a dedicated facility (Bio-Fab Research, Roma, Italy). Obtained sequences were compared with the JCPyV reference strain (GenBank: AB081613). Sequence alignment was performed using ClustalW2 [53] on the European Molecular Biology Laboratory–European Bioinformatics Institute (EMBL-EBI) website using default parameters.

Risk stratification was performed by using an anti-JCV index level, using a commercially available enzyme-linked immunosorbent assay (ELISA), considering 1.5 as cutoff value. Consequently, three risk categories, low (≤0.9), intermediate (0.9 < JCV index > 1.5) and high (>1.5) were distinguished. The anti-JCPyV index was only determined for the MS patients, not for HCs.

### 4.3. Viral miRNAs Investigation

Total RNA, including miRNA, was extracted from 200 μL of plasma or urine using the miRNeasy kit (Qiagen) according to the manufacturer’s instructions. MiRNA expression was analyzed and quantified with the specific JCPyV miR-J1-3p-5p quantitative TaqMan real-time PCR, as described previously [54]. Each reaction was carried out in triplicate with 15 ng of RNA and included negative controls. Synthetized oligonucleotides were used as standards (dilution range: 10^1^–10^6^ copies). The lower limit of detection was 10 copies of viral miRNA per ng of RNA. Human let7 miRNA was used as a control for endogenous miRNA expression in each assay [33,55]. One hundred nanograms of the JCPyV DNA of selected samples was PCR-amplified employing primers specific for the miRNAs expressing region, purified using the PCR Purification Kit (Qiagen, Milan, Italy) and sequenced in a dedicated facility (Bio-Fab Research, Rome, Italy). Obtained sequences were aligned and analyzed using ClustalW algorithm in BioEdit 7.2 (Tom Hall of Ibis Therapeutics, Carlsbad, CA, USA), applying default parameters.

### 4.4. Statistical Analysis

The results were analyzed with the use of the statistical method. The continuous variables are expressed both as mean ± SD and as median and range. All studied features were analyzed with non-parametric tests, such as the Friedman chi-square test, Kruskal–Wallis test, and Mann–Whitney U for unmatched data. This was due to the result of a Shapiro–Wilk test that showed a non-normal distribution of data. Specifically, differences in the relative occurrence of viral miRNAs between groups were assessed using a Fisher’s test. Differences between groups were considered statistically significant at *p* < 0.05. Differences in miRNA levels between groups were assessed using a Mann–Whitney test. Correlation between different parameters was analyzed using linear regression. The statistically significant *p*-value level was set at <0.05.

## 5. Conclusions

PML is a debilitating disease caused by JCPyV and for which there is no convincingly effective therapy. The size of the at-risk population is increasing and includes MS patients being treated with DMTs.

Adding to the problematic nature of the PML are the difficulties encountered in the diagnosis of this disease and the lack of useful *biomarkers* for PML progression.

In this study, for the first time, different approaches that aim at a rapid and early identification of JCPyV reactivation and possible onset of PML were discussed and analyzed together. Results emphasize that the molecular monitoring of JCPyV may allow early identification of patients at risk for PML among MS population and underline that the evaluation of the immunological status alone is not sufficient to limit the risk of PML. JCPyV miRNAs could be strongly proposed as *biomarkers* since they are stable, robustly detected, and their levels in body fluids may reflect viral perturbations in tissue sites.

## Figures and Tables

**Table 1:** JCPyV viruria, viremia, anti-JCV index and miRNAs expression: longitudinal investigation in multiple sclerosis patients (MS).

*T0*											
	*Viruria*		*Viremia*		*Anti-JCV Index*			*Urinary miR-J1-5p*		*Plasmatic miR-J1-5p*	
	*n (%)*	Load *	*n (%)*	Load *	*>1.5, n (%)*	<0.9–>1.5 *n* (%)	*<0.9, n (%)*	*n (%)*	Copies **	*n (%)*	Copies **
***OCRE***	6/19 (31.5)	5.5 × 10^2^	0/19 (0)	-	0/19 (0)	0/19 (09	19/19 (100)	15/19 (79)	980	15/19 (79)	900
***NAT***	10/19 (52.6)	5 × 10^3^	0/19 (0)	-	0/19 (0)	2/19 (10.5)	17/19 (89.5)	9/19 (47.4)	1100	9/19 (47.4)	980
***F***	1/2 (50)	3.5 × 10^3^	0/2 (0)	-	0/2 (0)	0/2 (0)	2/2 (100)	2/2 (100)	1000	2/2 (100)	900
***DMF***	1/2 (50)	3.0 × 10^3^	0/2 (0)	-	0/2 (0)	0/2 (0)	2/2 (100)	0/2 (0)		1/2 (50)	800
***TOT***	18/42 (43)		0/42 (0)		0/42	2/42 (4.8)	40/42 (95.2)	26/42 (62)		27/42 (64.3)	
***T6***											
	***Viruria***		***Viremia***		***Anti-JCV Index***			***Urinary miR-J1-5p***		***Plasmatic miR-J1-5p***	
	***n (%)***	Load *	***n (%)***	Load *	***>1.5, n (%)***	**<0.9–>1.5 *n* (%)**	***<0.9, n (%)***	***n (%)***	Copies **	***n (%)***	Copies **
***OCRE***	10/19 (52.6)	1 × 10^3^	0/19 (0)	-	5/19 (26.3)	5/19 (26.3)	9/19 (47.4)	15/19 (79)	820	15/19 (79)	730
***NAT***	18/19 (94.7)	7 × 10^5^	3/19 (15.8)	1.5 × 10^3^	12/19 (63.2)	6/19 (31.6)	1/19 (5.3)	7/19 (36.8)	850	7/19 (38.8)	700
***F***	1/2 (50)	5.5 × 10^4^	0/2 (0)	-	1/2 (50)	1/2 (50)	0/2 (0)	1/2 (50)	770	1/2 (50)	700
***DMF***	1/2 (50)	5 × 10^4^	0/2 (0)	-	1/2 (50)	1/2 (50)	0/2 (0)	0/2 (0)		0/2 (09	
***TOT***	30/42 (71.4)		3/42 (7.1)		19/42 (45.2)	13/42 (31)	10/42 (23.8)	23/42 (54.8)		23/42 (54.8)	
***T12***											
	***Viruria***		***Viremia***		***Anti-JCV Index***			***Urinary miR-J1-5p***		***Plasmatic miR-J1-5p***	
	***n (%)***	Load *	***n (%)***	Load *	***>1.5, n (%)***	**<0.9–>1.5 *n* (%)**	***<0.9, n (%)***	***n (%)***	Copies **	***n (%)***	Copies **
***OCRE***	19/19 (100)	6 × 10^4^	1/19 (5.3)	1 × 10^3^	10/19 (52.6)	7/19 (36.8)	2/19 (10.5)	12/19 (63.2)	520	12/19 (63.2)	540
***NAT***	19/19 (100)	8.5 × 10^6^	3/19 (15.8)	2.0 × 10^5^	15/19 (79)	2/19 (10.5)	2/19 (10.5)	5/19 (26.3)	500	5/19 (26.3)	570
***F***	2/2 (100)	1.5 × 10^5^	1/2 (50)	5.5 × 10^3^	1/2 (50)	1/2 (50)	0/2 (0)	1/2 (50)	490	1/2 (50)	510
***DMF***	2/2 (100)	1.0 × 10^5^	1/2 (50)	7.5 × 10^3^	1/2 (50)	1/2 (50)	0/2 (0)	0/2 (0)		0/2 (0)	
***TOT***	42/42 (100)		6/42 (14.3)		27/42 (64.3)	11/42 (26.2)	4/42 (9.5)	18/42 (43)		18/42 (43)	

Ocre: ocrelizumab; NAT: natalizumab; F: fingolimod; DMF: dimethyl-fumarate; TOT: total; T0: before starting treatments, no drugs administered; T6: 6 months after the beginning of therapy; T12: 12 months after the beginning of therapy; *n*: number of MS patients; %: percentage; * load: copies/mL; ** copies: copies/ng.

**Table 2:** JCPyV viruria, viremia, anti-JCV index and miRNAs expression: longitudinal investigation among healthy control subjects (HCs).

	*Viruria*		*Viremia*		*Urinary miR-J1-5p*		*Plasmatic miR-J1-5p*	
	*n (%)*	Load *	*n (%)*	Load *	*n (%)*	Copies **	*n (%)*	Copies **
T0	10/25 (40)	2.5 × 10^3^	0/25 (0)	-	8/25 (32)	800	16/25 (64)	1000
T6	8/25 (32)	5 × 10^3^	0/25 (0)	-	8/25 (32)	550	15/25 (60)	600
T12	9/25 (36)	3 × 10^3^	0/25 (0)	-	8/25 (32)	800	16/25 (64)	1000

HCs: healthy control subjects; T0: before starting treatments, 0 administration; T6: 6 months after the beginning of therapy; T12: 12 months after the beginning of therapy; *n*: number of HCs; %: percentage; * load: copies/mL; ** copies: copies /ng.

**Table 3:** Demographics and disease characteristics of MS patients and HCs.

	*MS Patients*			*HCs*
Variables	Baseline (T0)	T6	T12	
F/M	24/18	24/18	24/18	12/13
Median age in years (IQR)	31.5 (25.2–37)	33 (28.5–40)	34 (29.5–40.5)	34 (29.5–40.5)
Median years of disease (IQR)	5.5 (1.25–9.0)	6.5 (2–12)	7.5 (3–13)	N/A
Median EDSS (IQR)	2 (1.25–2.75)	2 (1–3)	2 (1.75–3)	N/A
**Therapy**	**Baseline (T0)**	**T6**	**T12**	N/A
Ocrelizumab (/n)	19/42	19/42	19/42	N/A
Natalizumab (/n)	19/42	19/42	19/42	N/A
Fingolimod (/n)	2/42	2/42	2/42	N/A
DMF (/n)	2/42	2/42	2/42	N/A

F: female; M: male; IQR: interquartile range; HCs: health control subjects; N/A: not applicable; EDSS: Expanded Disability Status Scale with value range from 0 (normal neurological examination) to 10 (bedridden patient) [49].

## Data Availability

Data are contained within the article.
